# Comparison of effects of aminosalicylic acid, glucocorticoids and immunosuppressive agents on the expression of multidrug-resistant genes in ulcerative colitis

**DOI:** 10.1038/s41598-022-19612-8

**Published:** 2022-11-30

**Authors:** Yan Chen, Ping Wang, Yin Zhang, Xiao-Yu Du, Ying-Jian Zhang

**Affiliations:** 1 Department of Gastroenterology, The First Affiliated Hospital, College of Clinical Medicine of Henan University of Science and Technology, No. 24 Jinghua Road, Luoyang, 471003 Henan China; 2 Department of Public Health, School of Medicine, Henan University of Science and Technology, Luoyang, 471003 Henan China

**Keywords:** Diseases, Gastroenterology, Molecular medicine

## Abstract

To compare the effects of aminosalicylic acid, glucocorticoids and immunosuppressants on the expression levels of multidrug resistance genes in patients with ulcerative colitis (UC), with the aim of providing a theoretical and therapeutic basis for the diagnosis, treatment, and prevention of UC. Fresh colonic mucosal tissues or postoperative pathological biopsies from 148 UC patients were collected, and the distribution sites and morphology of P-glycoprotein (P-gp) were detected using immunohistochemical staining. RT-PCR was used to quantify the expression levels of multidrug resistance gene (MDR1) mRNA before and after the corresponding treatment, and the effects of aminosalicylic acid, glucocorticoids and immunosuppressive drugs on P-gp were compared. In addition, the effects of the three drugs on MDR1 mRNA were analyzed. Administration of 5-aminosalicylic acid (5-ASA) drugs did not correlate with MDR1 expression in UC, whereas administration of glucocorticoids and immunosuppressive drugs was positively correlated with MDR1 expression profile. The expression levels of MDR1 mRNA and its product P-gp were significantly upregulated in patients who did not respond to glucocorticoids and immunosuppressive drugs. 5-ASA had no effect on the expression levels of MDR1 and its product P-gp in patients with a confirmed diagnosis of UC. However, the use of glucocorticoids and immunosuppressants can increase the expression level of MDR1.

## Introduction

Currently, there are no effective treatment options for ulcerative colitis (UC). Available pharmacological treatments are mainly aimed at reducing the severity of the associated symptoms. Commonly used drugs include aminosalicylic acid, corticosteroids, and immunosuppressive agents. The main aminosalicylates are sulfasalazine (SASP), which is cleaved to 5-aminosalicylic acid (5-ASA) and sulfasalazine (SP) by the action of azo reductase in the normal intestinal flora after oral administration. Among them, 5-ASA is the main active ingredient in drug therapy^1,2^. SASP is usually utilized to treat mild and moderate UC and needs to be combined with other types of drugs in the treatment of severe UC. New salicylic acid drugs are widely used in clinical practice. Another aminosalicylic acid drug, azo-salicylic acid, utilizes in the drug structure two molecules with azo linkage of 5-ASA. It reaches the end of the ileum after being administered orally, interacts with colonic bacteria in the stomach and small intestine, and is broken down by enzymes within 2 min. In addition, 5-ASA is more easily tolerated, although it can be equally effective as SASP, which can therefore be used as an alternative after 5-ASA resistance, with fewer side effects and better tolerability. The commonly used oral extended-release dosage forms of 5-ASA, including Pentasa, Asacol, Salofak, and Rowasa, are not significantly better than SASP^3^.

In addition to aminosalicylates, azathioprine is widely used in the treatment of inflammatory bowel disease, especially in hormone resistant or dependent patients with refractory UC and in patients with concomitant fistulas. Azathioprine exerts both immunosuppressive and immunomodulatory effects in order to block the proliferation and activation of T cells and to inhibit the chemotaxis of neutrophils. In addition, azathioprine reduces the dose of glucocorticoids in drug combination regimens and prolongs the remission period^4^. It exerts a drug effect that lasts 3 to 4 months, whereas glucocorticoids in combination take approximately 6 weeks to reduce the dose. During azathioprine dosing, patients should be monitored regularly every 2–3 months for white blood cell counts as well as thiopurine S-methyltransferase (TPMT) activity to predict the risk of myelosuppression after dosing^5^.

Inflammation is an adaptive response of the colonic mucosal immune system to the invasion of intestinal antigens. Under normal circumstances, the inflammatory response resolves immediately after the pathogen is cleared. If the inflammatory response does not resolve, it can deteriorate into a refractory chronic inflammatory bowel disease^6–11^. The aim of biologic therapy is to block inflammatory signaling pathways and inflammatory processes in the mucosa. Biotherapeutic agents include recombinant peptides, antibodies, nucleic acids (antisense oligonucleotides), etc. In recent years, tumor necrosis factor (TNF) inhibitors, such as infliximab, have been shown to produce positive results.

Glucocorticoids are anti-inflammatory and suppress the immune response and are one of the main drugs used in the treatment of UC. Clinically glucocorticoids are mainly used to treat patients in whom aminosalicylic acid is ineffective, as well as in patients with acute UC. It exerts powerful anti-inflammatory effects and rarely produces systemic adverse effects^12^. The representative drug, budesonide, is rapidly inactivated in the liver after intestinal absorption and has no effect on serum cortisol levels. Long-term use of such drugs is prone to side effects and does not prevent relapse. Therefore, glucocorticoid dosage should be reduced until complete discontinuation of the drug after the associated symptoms have been relieved^12^.

Because UC often undergoes recurrent, alternating periods of activity and remission, the dosing cycle is also long. Drug resistance is inevitable during this dosing process, and it greatly affects patient regression. Despite the considerable progress in drug therapy and the expansion of treatment options in the last decade, treatment resistance remains a major problem. Biomarkers such as oncostatin M can identify patients who may be resistant to anti-TNF and other traditional IBD therapies^13^; however, alternative treatment options have been lacking due to the lack of a clearly defined resistance mechanism.

The MDR1 gene is an attractive candidate for the pathogenesis of UC, perhaps in response to therapy, with evidence at both the functional and genetic levels. Its product, P-glycoprotein (P-gp), is a transmembrane efflux pump, thereby influencing the metabolism and response to many drugs, some of which (e.g., glucocorticoids) are central to IBD therapy. p-gp binds both drugs and ATP, and with the help of ATP supply, P-gp allows intracellular drugs to be pumped out of the cell, diminishing the intracellular drug concentration making the cell resistant to the drug This reduces the intracellular drug concentration and makes the cells resistant to the drug.

P-gp plays a very important role in ADME processes (absorption, distribution, metabolism, excretion) and drug-drug interactions (DDI) in humans. In addition, P-gp is highly expressed on many epithelial surfaces, including the gastrointestinal tract (G-I), and has a putative role in reducing the absorption of endogenous or exogenous toxins, and perhaps host-bacteria interactions. Thus, there is an urgent need to elucidate how the MDR1 gene and its product P-gp are affected during the long-term treatment of UC.

In this multicenter study, 148 patients with UC were selected from outpatients and inpatients in three hospitals and treated with different types of drugs. The patients were also followed up and the corresponding tissue samples were collected to generalize the effects of aminosalicylic acid, glucocorticoids and immunosuppressants on the expression levels of multidrug resistance genes in UC patients, aiming to provide a theoretical basis for the diagnosis and treatment with drugs in UC.

## Materials and methods

### Study subjects

A total sample of 148 UC patients (study group), 83 males and 65 females, aged 16–69 years (mean age: 42.40 ± 13.21 years), outpatients and inpatients of the First, Second and Third Affiliated Hospitals of Henan University of Science and Technology from March 2015 to December 2019 was obtained. Among them, 58 were treated with ASA drugs, 53 with glucocorticoids and 37 with immunosuppressive drugs. Forty-five patients, 25 males and 20 females, aged 18–65 years, with a mean age of (36.70 ± 11.41) years, who underwent e-colonoscopy suggestive of no intestinal symptoms, had a history of irritable bowel syndrome (IBS) or UC, and had no history of drug allergy or use of amino acid/immunosuppressive drugs or use of glucocorticoid drugs were enrolled in the control group. All patients were followed up from the admission or discharge date until December 2019. Colonoscopic biopsy and postoperative pathology were considered as the gold standard. General data were compared as shown in Table 1.

**Table 1:** Comparison of general data.

Grouping	Case number (*n*)	Gender (*n*,%)		Age (years, (*X* ± *S*))
		Male	Female	
Experimental group	148	83 (56.08%)	65 (43.92%)	42.40 ± 13.21
Control group	45	25 (55.56%)	20 (44.44%)	36.70 ± 11.41
(^2^/*F* value		1.213	1.737	
*P* value		0.749	0.165	

### Classification and staging of UC

Patients with incomplete information were excluded from the follow-up trial. All enrolled patients were classified as mild and severe according to the "Chinese Consensus on the Diagnosis and Treatment of Inflammatory Bowel Disease" (hereinafter referred to as "07 Consensus")^14^, which was developed by the Chinese Society of Gastroenterology in 2007, as shown in Table 2.

**Table 2:** Truelove-Witts Severity Index for UC.

Item	Mild	Severe
Excrement (time/day)	< 4	> 6
Hematochezia	Light or no	Severe
Body temperature (℃)	Normal	> 37.5
Pulse (time/min)	Normal	> 90
Hemoglobin	Normal	> 75%
Erythrocyte sedimentation rate (mm/1 h)	< 30	> 30

According to the course of UC, UC can be divided into initial, chronic relapsing, chronic persistent and acute fulminant forms. In addition, according to the disease stage, UC can be divided into active and remitting stages. The diagnosis was made by reference to the Sutherland Disease Activity Index (DAI). If the index score was below 2, it was classified as remission; if it was above 2, it was classified as active, as shown in Table 3.

**Table 3:** Sutherland disease activity index.

Item	Scoring			
	0	1	2	3
Diarrhea hemafecia	Normal	1–2 time/d	3–4 times/d	5–6 times/d
	No	A little	Obvious	Give priority to with blood
Mucous membrane performance	Normal	Mildly brittle	Moderately brittle	Severely brittle with exudation
The physician evaluates the condition	Normal	Mild	Moderate	Severe

A total score below 2 was considered symptom relief; between 3 and 5 was mild activity; 6–10 was moderate activity; 11–12 was severe activity.

### Lesion site

The lesion sites can be further classified as rectum, left hemicolectum, wide colon and total colon. The left hemicolon referred to the flexure of the spleen of the colon toward the rectum. The wide colon included the splenic flexure on the proximal end of the colon. Lesions confined to the splenic flexure of the distal colon were classified as left hemicolon lesions, and those beyond the splenic flexure but not to the extent of total colon lesions were considered wide colon.

### Diagnostic criteria

The diagnosis of UC can be confirmed by clinical manifestations, such as diarrhea, mucopurulent blood, with abdominal pain, defecation and various degrees of systemic symptoms; persistent, recurrent episodes; duration of UC > 1 month, colonoscopic observation of distal colon such as rectum and sigmoid colon with continuous, diffuse distribution, mucosal vascular congestion, hemorrhage and blurred texture, etc. 1 month, colonoscopic observation of rectum, sigmoid colon and other distal colon with continuous Diffuse distribution, mucosal vascular congestion, hemorrhage, edema, blurred, disorganized or absent texture, purulent secretion adhesions, barium enema examination including jagged or burr-like intestinal margins, multiple small filling defects in the intestinal wall, coarse mucosa with/ or granular changes, shortening of the intestine, disappearance of the intestinal pouch into a lead tube, pathological examination. Exclusions were those with bacillary dysentery, amebic dysentery, chronic schistosomiasis, intestinal tuberculosis and other infectious colitis, colonic Crohn's disease (CD), ischemic colitis, and radiation colitis.

### Inclusion criteria

Those who did not receive ASA medication, oral or intravenous glucocorticoids, glucocorticoids plus immunosuppressants and herbal medicine 4 weeks before the start of the study; those who received oral ASA medication after treatment: oral or intravenous glucocorticoids; oral or intravenous glucocorticoids plus immunosuppressants, ASA medication and glucocorticoids *and other* topical treatment medications.

### Prognosis and outcome

All patients were divided into effective (complete remission and effective), ineffective and control groups according to their prognosis and efficacy. In the complete remission group, the patients' symptoms disappeared, and the mucosa was normal on colonoscopy. In the effective treatment group, the patients' physical symptoms of colitis largely disappeared, and diarrhea and abdominal pain decreased; colonoscopy showed mild inflammation of the mucosa or pseudo-polyps formation; pathological sections showed restoration of the mucosal layer of colonic tissue and reduction of neutrophil infiltration.

In the ineffective treatment group, patients had no relief of symptoms after treatment and frequent diarrhea and abdominal pain; colonoscopy showed severe inflammation of the mucosa and breakdown of the mucosal layer; pathological sections showed defects in the mucosal layer of colonic tissue and increased neutrophil infiltration.

### Specimen preparation

Fresh colonic mucosal tissue specimens or endoscopic pathological mucosal tissues or postoperative pathological biopsies were immediately stored in a refrigerator at − 80 °C for RNA extraction. The remaining specimens were adequately fixed in 10% formalin, routinely dehydrated, and embedded in paraffin, and 4-μm tissue sections were prepared for immunohistochemical staining (P-gp).

### Immunohistochemical staining

#### Immunohistochemical staining method

Using the SP method, antigen repair was required for the determination of P-gp. The negative control was replaced with phosphate-buffered saline (PBS) buffer, and the rest of the procedure was performed according to the manufacturer's instructions. p-gp positive control was provided by Beijing Zhongshan Biotechnology Co. Positive controls were set up for each antibody. Routine dewaxing was hydrated using xylene twice for 15 min each, hydrated with gradient alcohol, washed three times with PBS (pH = 7.4) for 5 min each, and incubated with 3% hydrogen peroxide solution for 15 min at room temperature to eliminate endogenous peroxidase. Then, the samples were washed with distilled water for 5 min for three times. For antigen repair, 10 ml of ethylenediaminetetraacetic acid (EDTA) antigen repair solution was collected and diluted with 490 ml of triple distilled water for 3–4 min of hot repair. Finally, samples were cooled to room temperature, washed three times with PBS for 5 min each, sealed with normal goat serum working solution, and incubated at 37 °C for 40 min. The primary antibody was added to the wet box overnight at 4 °C, rewarmed at 37 °C for 45 min, and washed three times with PBS for 5 min each. The labeled secondary antibody was added to biotin drops and incubated at 37 °C for 25 min, washed three times with PBS, and horseradish peroxidase-labeled streptomycin was incubated at 37 °C for 20 min, washed three times with PBS for 5 min. The color development reaction was observed under a microscope with a chromogenic solution of 3,3-N-diaminobenzidine tetrahydrochloride (DAB). Samples were rinsed with running water, gently re-stained with hematoxylin for 10–30 s, dehydrated with a gradient of alcohol, clear xylene, and then mounted with a neutral mounting sheet.

#### Evaluation of immunohistochemical staining results

Using DAB immunohistochemical color development system, positive results were shown as brownish-yellow particles in the corresponding positions. p-gp positive particles were mainly distributed in the lamina propria of colonic mucosa and intestinal epithelium, and a distinct brownish-yellow color in the membrane and cytoplasm was considered positive. Ten pathological cells were randomly counted in the high magnification field. the number of P-gp treated positive cells was < 10% as negative, 10–25% as (+) and 25–75% as (+++). This refers to the criteria provided by Zhongshan Biotechnology Co.

### Reverse transcription polymerase chain reaction (RT-PCR)

#### Primer

Primers designed by Primer Premier 5.0 were used as reference^15^. Primers for MDRI and β-actin were synthesized by Beijing Sebring Bioengineering Co.

- MDR1-specific primers.
  - Forward: 5′-AGATCAACTCGTAGGAGTGTC-3′.
  - Reverse side: 5′-GTTTCTGTATGGTACCTGCAA-3′.

- β-actin primers.
  - Forward: 5'-GAGACCTTCAACCCCAGCC-3'.
  - Reverse: 5'-GGCCATCTCTTGCTCGAAGTC-3'.

#### Extraction of total tissue RNA

Fresh specimens collected by colonoscopic biopsy or post-surgical tissue biopsy were immediately stored at − 80 °C in a low-temperature refrigerator to detect the expression level of MDR1.

#### Trizol extraction^16^

Harvest frozen tissue samples and grind to a powder and transfer to pre-cooled 1.5 ml EP tubes before evaporation of liquid nitrogen. Add LML Trizol, insert into a microtissue homogenizer for 2–3 min, then add chloroform and shake for 15 s, allow to stand for 5 min, then centrifuge at 12,000 rpm for 15 min at 4 °C. The aqueous component of the upper layer was carefully collected into another new EP tube. Pre-chilled isopropanol at − 20 °C was added and mixed thoroughly with the supernatant phase. After 10 min of ice bath and centrifugation at 12,000 rpm for 15 min, the supernatant was discarded and dried, 1 ml of 75% ice ethanol was added and mixed well, the precipitate was washed well and centrifuged at 8000 rpm for 5 min at 4 °C, then the supernatant was discarded and the precipitate was dried. If to be used immediately, leave the sample at ambient temperature for 20 min and add 30 μl of DEPC water. If not for immediate use, add 75% ethanol LML and store at − 80 °C.

#### RNA quality identification

**Figure 1:**
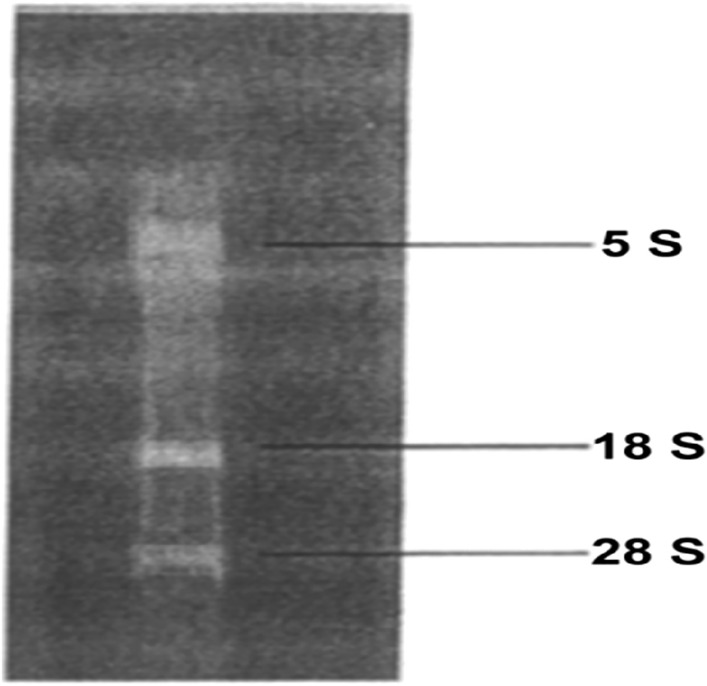
Total RNA integrity test.

The purity of RNA was good (OD_260_ /OD_280 _≈ 1.97) under OD_260_ and OD_280_ of ULTRAVIOLET spectrophotometer. The 5S, 18S and 28S bands were clearly visible by formaldehyde gel electrophoresis, and the extracted RNA was not degraded, as shown in Fig. 1.

#### RT-PCR

We used a two-step RT-PCR for quantification. The first step was cDNA reverse transcription. Vortex and shake the reagent solution from the melted kit for 2 s; transient centrifuge in a micro benchtop centrifuge for 5 s; transfer 4.5 µl of the bootstrap solution to a new 0.5 ml PCR tube; add 8 µl of RNA sample (5 µg of total RNA or 0.25 µg of POLY A + RNA); mix with a gun tip; incubate in a dry thermostat at 70 °C for 3 min; immediately place in an ice bath; add 8 µl of Reverse Solution (containing Invitrogen SuperScript III Reverse Transcriptase); mix well with the tip of the gun; centrifuge instantaneously for 5 s in a micro benchtop centrifuge; incubate for 60 min at 37 °C in a dry thermostat; place immediately in an ice bath; dilute with 80 µl of buffer and mix well; place in an ice bath or place in a − 20 °C refrigerator. Storage.

The second step was cDNA amplification. Transfer 30 µl of reaction solution to a new 0.5 ml PCR tube; add primers F and R (350 ng total, respectively) and Taqase; add 2 µl of the above diluted cDNA synthesis; finally add buffer to a total of 50 µl and mix well; place in a 4 °C micro benchtop centrifuge for 5 s instantaneously; add 2 drops of mineral oil using a 1000 µl gun tip. The reaction was carried out in a PCR amplifier and the products were later subjected to agarose electrophoresis for characterization.

PCR reaction conditions: pre-denaturation at 94 °C for 5 min. Cycling parameters: 94 °C for 1 min, 50 °C for 1 min, 72 °C for 1 min, 35 cycles, extension for 5 min.

### Identification of amplification products

A 2% agar gel (containing ethidium bromide 0.5 μl/ml) was prepared with TAE, and 10UL amplification products were sampled by electrophoresis at 100 V for 120 min. The amplification products of MDRl and B-actin were identified under UV light at 167 bp and 301 bp, respectively, which were consistent with the designed amplification fragments of MDR1 and P-actin primers.

#### Quantitative analysis

Two bands of MDRl and B-actin primers were scanned with a Cs-910 chromatography scanner made by Shimadzu. The length and width were 2.1 mm and 0.1 mm. The wavelength input was 550 nm. The data were entered into a computer for relative quantitative analysis, and MDR1/B-actin was the relative amount of MDR1.

### Statistical analysis

The SPSS 19.0 statistical package was used for statistical analysis (SPSS Inc., Chicago). Percentages were compared using the Chi-squire test, and t-tests were used for comparison of measurements between the two groups. p-values less than 0.05 were considered statistically significant.

### Ethics approval and consent to participate

This study was approved by the ethics committee of the first affiliated hospital, and college of clinical medicine of henan university of science and technology. Informed consent for all patients (including the patients of the control group) was obtained prior to therapy and performed in accordance with the Declaration of Helsinki and Good Clinical Practice Guidelines.

## Results

### Results of immunohistochemical staining for P-gp in colon tissue

**Figure 2:**
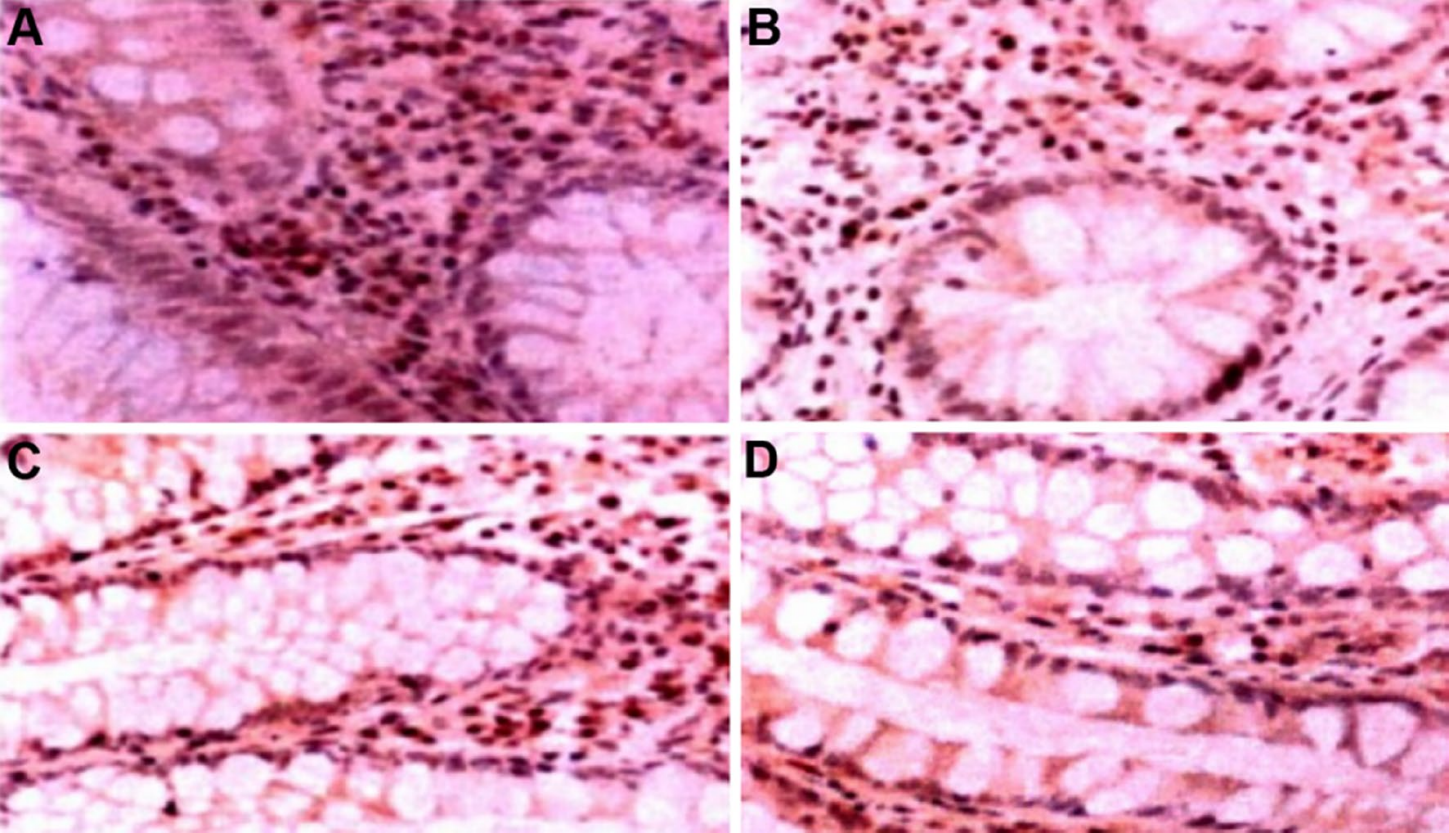
Detection results of P-gp immunohistochemical staining. (**A**) Results of P-gp staining in epithelial cells of healthy control colon tissues. (**B**) Results of P-gp staining in the lamina propria of healthy control colon tissue. (**C**) Results of P-gp staining in the epithelial cells of colon tissue of UC patients. (**D**) Results of P-gp staining in the lamina propria of colonic tissue from UC patients. Magnification is 400x.

The particles positive for P-gp staining were mainly distributed in the lamina propria of colonic mucosa and intestinal epithelial cells, which showed a distinctive brownish-yellow color in the cell membrane and cytoplasm. In the control group, P-gp stained positively in the epithelial cells and lamina propria (Fig. 2A,B), and in the UC colonic mucosa P-gp stained positively in both epithelial and lamina propria cells (Fig. 2C,D).

### Effect of ASA on P-gp expression in colonic tissue of UC patients

**Figure 3:**
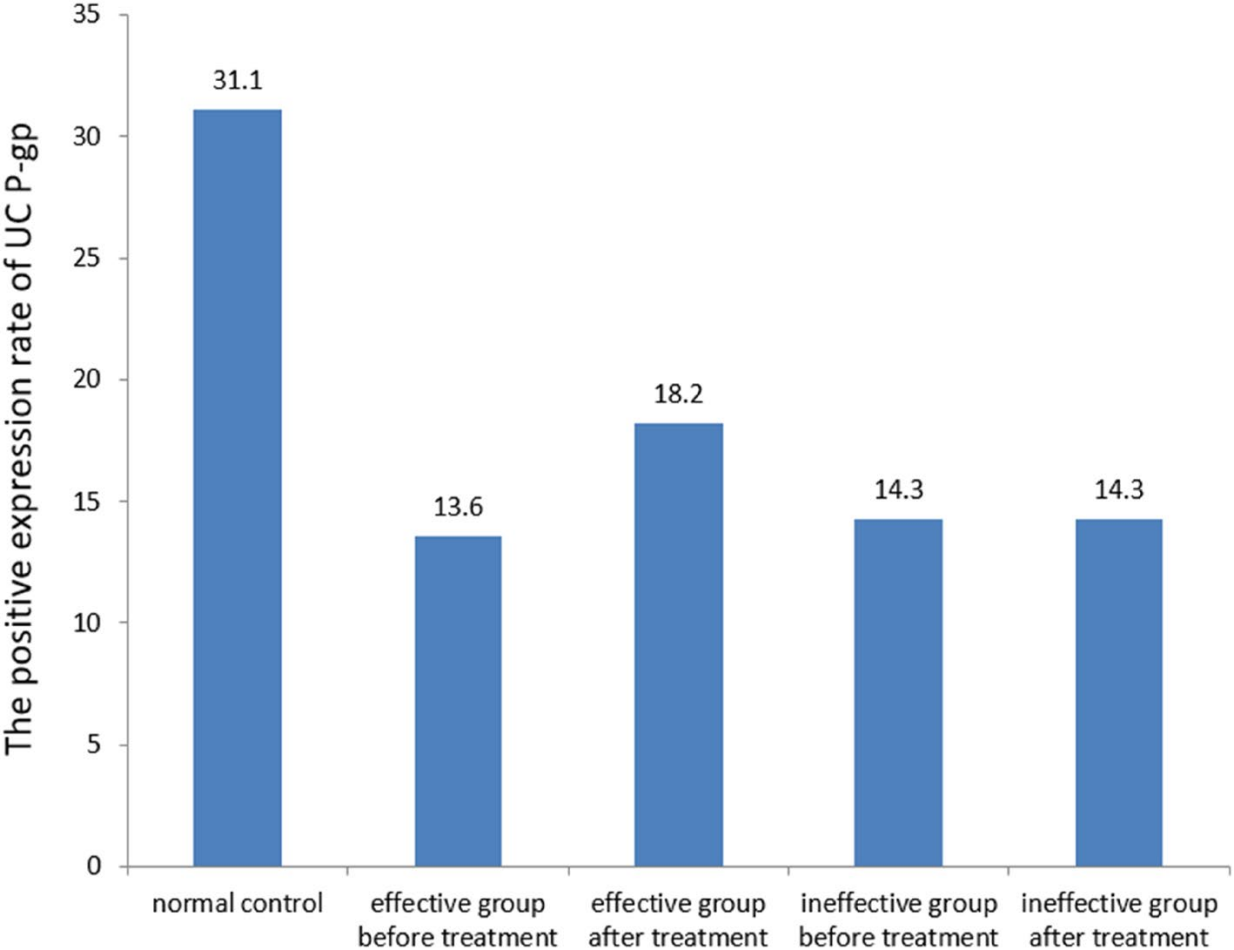
Effect of aminosalicylic acid drugs on P-gp expression in ulcerative colitis. Calculated from the data in Table 4, shown as mean values. Significant differences between groups are detailed in Table 4.

Among 58 UC patients treated with ASA, there was a significant difference in the expression rate of P-gp before and after treatment in the effective group of UC compared with the normal control group (P < 0.05). However, in the effective and ineffective groups, there was no significant difference in the positive expression rate of P-gp after treatment compared with that before treatment (all P > 0.05), as shown in Table 4 and Fig. 3.

**Table 4:** Effect of ASA drugs on the expression of P-gp in UC.

Group	Total (n)	Positive (n)	Negative (n)	Positive rate (%)
Control group	45	14	31	31.1
**Effective group**				
Pre-treatment	44	6	38	13.6^a^
Post-treatment	44	8	36	18.2^ab^
**Ineffective group**				
Pre-treatment	14	2	12	14.3^a^
Post-treatment	14	2	12	14.3^ab^

^a^*P* < 0.05 versus normal control.

^b^*P* > 0.05 versus pre-treatment.

### Effect of glucocorticoids on P-gp expression in colonic tissue of UC patients

**Figure 4:**
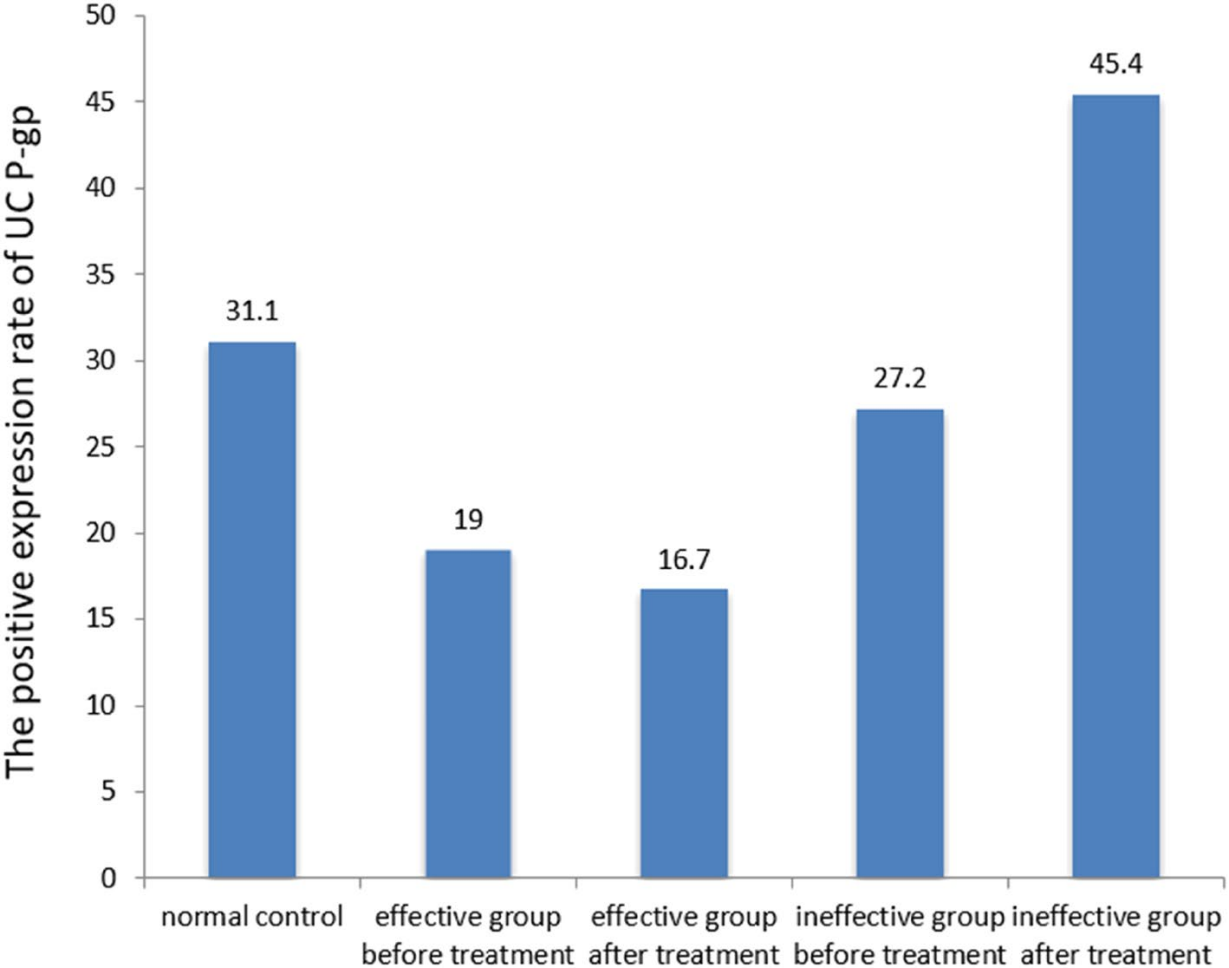
Effect of glucocorticoids on P-gp expression in ulcerative colitis. Calculated from the data in Table 5, shown as mean values. Significant differences between groups are detailed in Table 5.

In 53 patients treated with glucocorticoids, the rate of positive P-gp expression was significantly downregulated in the effective group compared with the control group, both before and after treatment (all P < 0.05). The P-gp positive expression rate in the inactive group was not significantly different before and after treatment (all P > 0.05). In the nulliparous group, the positive expression rate of P-gp was significantly higher than that of the control group after treatment (P < 0.05). As shown in Table 5 and Fig. 4, the positive expression rate of P-gp was significantly higher in the ineffective group than in the effective group before and after treatment (all P < 0.05).

**Table 5:** Effect of glucocorticoid on P-gp expression in UC.

Group	Total (n)	Positive (n)	Negative (n)	Positive rate (%)
Control group	45	14	31	31.1
**Effective group**				
Pre-treatment	42	8	34	19.0^a^
Post-treatment	42	7	35	16.7^ab^
**Ineffective group**				
Pre-treatment	11	3	8	27.2^d^
Post-treatment	11	5	6	45.4^ace^

^a^*P* < 0.05 versus control group.

^b^*P* > 0.05 versus pre-treatment.

^c^*P* < 0.05 versus pre-treatment.

^d^*P* < 0.05 versus effective group before treatment.

^e^*P* < 0.05 versus effective group post-treatment.

### Effect of immunosuppression on P-gp expression in UC

**Figure 5:**
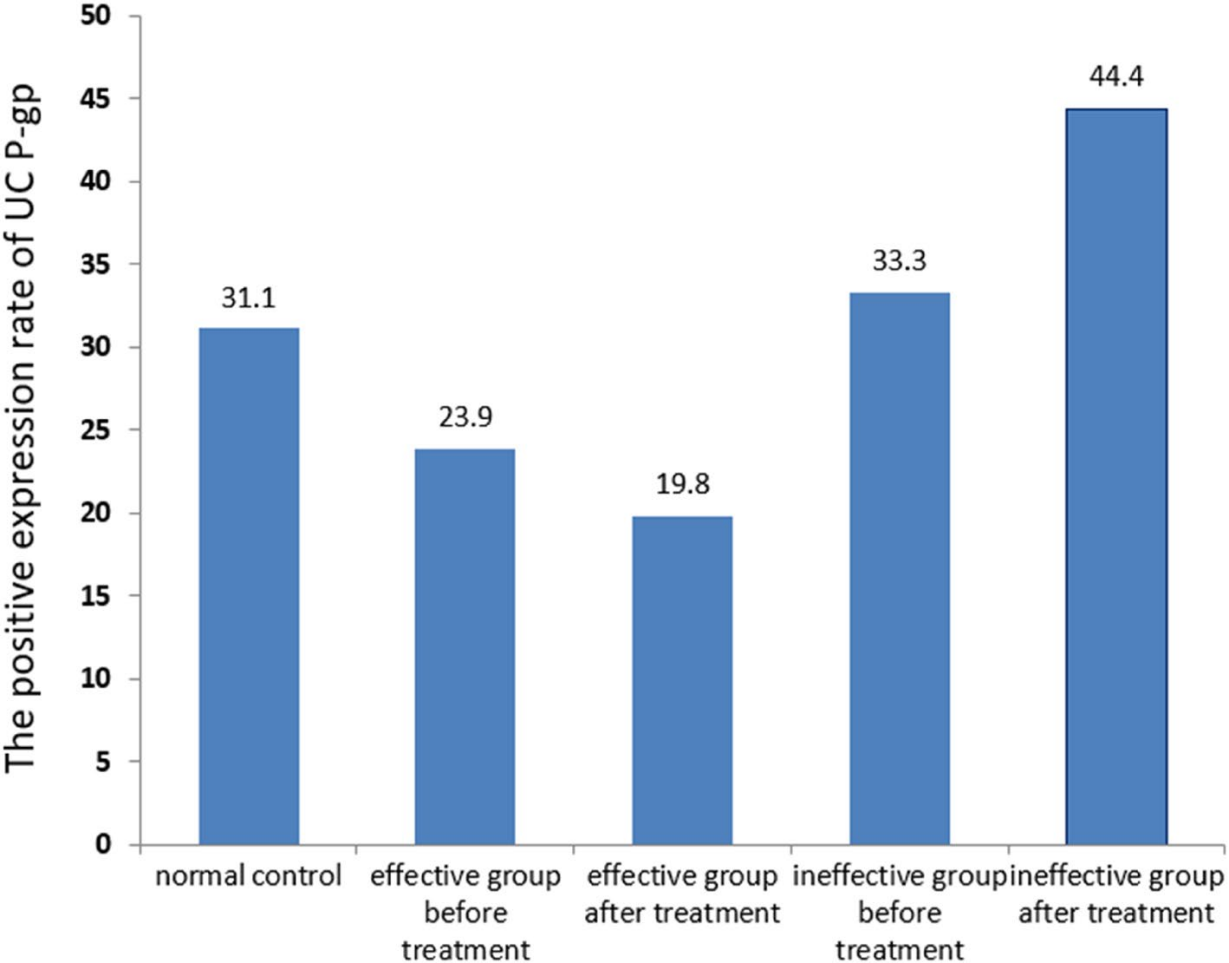
Effect of immunosuppression on P-gp expression in ulcerative colitis. Calculated from the data in Table 6, shown as mean values. Significant differences between groups are detailed in Table 6.

In 37 cases of UC with immunosuppression, the positive expression rate of P-gp was significantly lower in the effective group than in the control group before and after treatment (all P < 0.05). However, there was no significant difference in the positive expression rate of P-gp in the effective group before and after treatment (P > 0.05). The positive expression rate of P-gp before treatment did not differ between the ineffective and control groups (*P* > *0*.05), while it increased significantly after treatment compared with the control group (*P* < 0.05). In the effective group, the positive expression rate of P-gp was not significantly different before and after treatment (P > 0.05), as shown in Table 6 and Fig. 5.

**Table 6:** Effect of immunosuppressants on P-gp expression in UC.

Group	Total (n)	Positive (n)	Negative (n)	Positive rate (%)
Control group	45	14	31	31.1
**Effective group**				
pre-treatment	28	5	23	23.9^a^
Post-treatment	28	6	22	19.8^ab^
**Ineffective group**				
Pre-treatment	9	3	6	33.3^d^
Post-treaent	9	4	5	44.4^ace^

^a^*P* < 0.05 versus normal control.

^b^*P* > 0.05 versus pre-treatment.

^c^*P* < 0.05 versus pre-treatment.

^d^*P* < 0.05 versus effective group pre-treatment.

^e^*P* < 0.05 versus effective group post-treatment.

### Effect of ASA on MDR1 gene expression in UC

In 58 UC patients treated with ASA, the expression levels of MDR1 gene before and after treatment in the effective group were 0.590 ± 0.071 and 0.514 ± 0.018, respectively, which were significantly lower (both P < 0.05) compared with the control group (1.374 ± 0.022). However, the expression levels of MDR1 gene in the effective and ineffective groups after treatment were not significantly different from those before treatment (all P > 0.05), as shown in Table 7.

**Table 7:** Effect of ASA drugs on the expression level of MDR1 gene in UC (mean ± SD).

Group	Total (n)	Relative expression of MDR1
Control group	45	1.374 ± 0.022
**Effective group**		
Pre-treatment	44	0.590 ± 0.071^a^
Post-treatment	44	0.514 ± 0.018^ab^
**Ineffective group**		
Pre-treatment	14	0.454 ± 0.073^a^
Post-treatment	14	0.314 ± 0.062^a^

^a^*P* < 0.05 versus normal control.

^b^*P* > 0.05 versus pre-treatment.

### Effect of glucocorticoids on MDR1 gene expression in UC

The expression levels of MDR1 gene before and after treatment in the effective group were 0.675 ± 0.103 and 0.509 ± 0.106, respectively, which were significantly lower than that of 1.374 ± 0.022 in the control group (all P < 0.05). In the effective group, the expression levels of MDR1 gene were not significantly different before and after treatment (P > 0.05). The expression level of MDR1 in the inactive group was not significantly different from the control group before treatment (*P* > *0*.05), and the expression level was substantially upregulated after treatment (P < 0.05). The expression levels of MDR1 gene in the inactive group were significantly higher than those in the effective group before and after treatment (all *P* < *0*.05), as shown in Table 8.

**Table 8:** Effect of glucocorticoid on the expression level of MDR1 gene in UC (mean ± SD).

Group	Total (n)	Relative expression of MDR1
Control group	45	1.374 ± 0.022
**Effective group**		
Pre-treatment	42	0.675 ± 0.103^a^
Post-treatment	42	0.509 ± 0.106^ab^
**Ineffective group**		
Pre-treatment	11	1.249 ± 0.073^d^
Post-treatment	11	1.867 ± 0.151^ace^

^a^*P* < 0.05 versus control group.

^b^*P* > 0.05 versus pre-treatment.

^c^*P* < 0.05 versus pre-treatment.

^d^*P* < 0.05 versus effective group pre-treatment.

^e^*P* < 0.05 versus effective group post-treatment.

### Effect of immunosuppression on MDR1 gene expression in UC

The expression levels of MDR1 gene in the effective group before and after treatment were 0.618 ± 0.095 and 0.523 ± 0.0201, respectively, which were significantly different from 1.374 ± 0.022 in the control group (both P < 0.05). The expression levels of MDR1 gene in the effective group were not significantly different before and after treatment (P > 0.05). In the ineffective group, the expression levels were not significantly different before treatment compared with the control group (*P* > *0*.05), while they increased sharply after treatment (P < 0.05). The expression levels of MDR1 gene before and after treatment in the null group were statistically significant compared with the effective group (both *P* < *0*.05), as shown in Table 9.

**Table 9:** Effect of immunosuppressive agents on the expression of MDR1 gene in UC (mean ± SD).

Group	Total (n)	Relative expression of MDR1
Control group	45	1.374 ± 0.022
**Effective group**		
Pre-treatment	28	0.618 ± 0.095^a^
Post-treatment	28	0.523 ± 0.0201^ab^
**Ineffective group**		
Pre-treatment	9	1.263 ± 0.0833^d^
Post-treatment	9	1.885 ± 0.054^ace^

^a^*P* < 0.05 versus control group.

^b^*P* > 0.05 versus pre-treatment.

^c^*P* < 0.05 versus pre-treatment.

^d^*P* < 0.05 versus effective group pre-treatment.

^e^*P* < 0.05 versus effective group post-treatment.

## Discussion

The regulatory relationship between NF-κB and MDR1 has been the subject of several studies^17–19^. Ogretmen et al. demonstrated that a protein complex composed of NF-κB /p65 and c-Fos transcription factors interacts with the CAAT promoter region in MCF7 cells to negatively regulate human mdr1 promoter activity. It has also been reported that insulin-induced mdr1 expression is mediated by NF-κB in rat hepatoma cells^20^, NF-κB can protect renal proximal tubular cells from cadmium and oxidative stress by increasing the expression of P-gp. Recently, it has also been reported that NF-κB is involved in TNF-α-induced mdr1 expression in hepatocytes, in 2-acetylaminofluorene-induced MDR expression in hepatocytes and in constitutive MDR expression in drug-resistant cells^21^. Therefore, the effect of drugs on NF-κB during the treatment of inflammatory diseases like UC and whether it subsequently causes upregulation of MDR1 and increased drug resistance are questions that need to be addressed.

For the aminosalicylates, our immunohistochemical staining and RT-PCR assays showed that the expression of both P-gp and MDRl genes before and after treatment with aminosalicylates was significantly different from that of normal controls. However, there was no statistically significant difference in the expression level of P-gp after treatment compared with that before treatment in both the effective and ineffective groups. The above data suggest that MDR gene expression in colonic tissues of patients with ulcerative colitis has been changed before treatment, and drug treatment does not affect MDR gene expression, i.e., it does not change the increase in MDR gene resistance or decrease its expression. Therefore, aminosalicylates can be used for long-term pharmacotherapy of UC without causing pharmacogenic tolerance.

SASP is an NF-κB (P65) inhibitor with potent inhibitory effects on NF-κB (P65) activation. This has been confirmed by several studies. For example, SASP has been shown to inhibit cell proliferation by blocking NF-κB (P65) activation. And the downregulation of mRNA and protein levels of Bcl-2, Cyclin D1, MDRl and NF-κB (P65) during chemotherapy is one of the reasons for and MDR gene expression in pancreatic cancer^22^, suggesting that NF-κB negatively regulates the expression of MDR1.

For glucocorticoids, the expression levels of MDR1 gene and P-gp were significantly higher in the ineffective group after treatment compared to the pre-treatment and healthy controls. Not only that, the expression levels of MDR1 gene and P-gp in the ineffective group were significantly higher than those in the effective group. These results may be due to the ability of glucocorticoids to be exocytosed by P-gp.

Clinically, the effectiveness of UC treatment depends mainly on the patient's response to glucocorticoid therapy. For some patients, there is no response even after high doses of the drug, which leads to treatment delays^23^. P-gp can mediate glucocorticoid resistance through several pathways. First, P-gp can induce glucocorticoid resistance by acting on drug transport pumps and protecting cells from apoptosis. In addition, P-gp can also mediate hormone resistance by acting on the entire cellular immune system^24^. The induction of resistance by glucocorticoids during the treatment of UC has also been validated by several studies. In patients with UC without hormone therapy, P-gp expression and activity in peripheral blood, intestinal mucosal lamina propria and intestinal epithelial lymphocytes were significantly lower than in normal controls^25^. For those patients receiving hormone therapy peripheral blood mononuclear cells MDR1 mRNA expression showed a significant positive correlation with the total hormone dose, suggesting that the high expression of MDR1 mRNA in UC patients is a response induced by the application of high dose hormone therapy^26^.

For immunosuppressants, our study demonstrated that immunosuppressants can upregulate P-gp expression as well as glucocorticoids. It may be that lymphocytes act as a protective mechanism to specifically increase P-gp expression and reduce the toxic effects of drugs by external stimuli. fiedler also found that combined interventions such as glucocorticoids and spinosad also increased MDR gene as well as P-gp protein expression in rat liver and intestine. The above evidence suggests that MDR gene and P-gp protein expression are not only related to individual genetics, but that glucocorticoids and immunosuppressants themselves can also increase MDR gene and P-gp protein expression^27^. Therefore, although competitive substrate resistance reversal can improve drug efficacy in a short period of time, the high expression of MDR-induced genes and P-gp protein may be associated with drug resistance or drug dependence after long-term use, which may also be one of the reasons for relapse after drug administration in many diseases.

Other studies have similarly identified resistance induced by immunosuppressive therapy. For example, patients with lupus erythematosus with higher disease activity have increased MDR gene and P-gp protein expression in the presence of immunosuppressive agents such as cyclophosphamide and the glucocorticoid methylprednisolone^28^. Also, the different expression levels of P-gp in lymphocytes of SLE patients indicate a different requirement for immunosuppression^29^, which corroborates that immunosuppression can induce the development of drug resistance. More direct evidence is that intestinal MDR1 expression is also upregulated after tacrolimus administration^30^. Related mechanistic studies suggest that immunosuppression induces phosphorylation of AKT and ERK, leading to activation of PI3K/AKT and MAPK/ERK pathways and increased expression of MDR1/P-gp in target cells^31^. The relationship between MDR1/P-gp and the development of UC has also been continuously validated by mechanistic studies^32^.

In summary, the expression level of MDR1/P-gp must be precisely determined and monitored during the treatment of UC to avoid the increase of drug resistance caused by drug abuse, which reduces the efficacy.

## Conclusion

ASA had no significant effect on the expression level of MDR gene in UC patients. The use of glucocorticoids and immunosuppressants significantly increased the expression levels of MDR genes in the colonic tissues of UC patients, and the underlying mechanisms remain to be further elucidated.

## Data Availability

The datasets used and/or analyzed during the current study are available from the corresponding author on reasonable request.
